# High luminescent polymers for stretchable displays

**DOI:** 10.1093/nsr/nwac093

**Published:** 2022-05-18

**Authors:** Zhitao Zhang, Zhenan Bao

**Affiliations:** Department of Chemical Engineering, Stanford University, USA; School of Chemistry and Chemical Engineering, Shanghai Jiao Tong University, China; Department of Chemical Engineering, Stanford University, USA

## Abstract

This perspective summarizes the main approaches to realize stretchable displays with high performance as well as future directions.

Displays severing as the human*–*machine interface are playing indispensable roles in our daily lives. The machine displays information visually to allow the user to respond directly. However, traditional bulky and rigid displays cannot meet the requirements of the fast-developing wearable electronics, considering skin compatibility, portability and durability under exercise [1]. Next-generation displays should be soft and stretchable, so that they can be easily integrated with other types of stretchable electronic devices, intimately attaching onto or biocompatibly implanting into the human body for real-time sensing, monitoring and readout [2]. To achieve this goal, device engineering and rational material design have been recognized as two effective strategies. In this perspective, we will summarize the main approaches to realize stretchable displays with high performance as well as future directions (Fig. 1).

**Figure 1.**
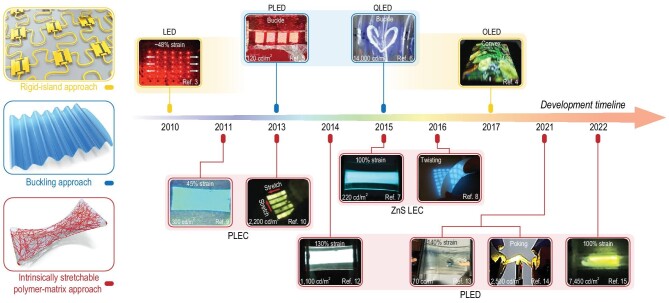
Timeline of developments in stretchable display materials. LED, light-emitting diode; PLED, polymer light-emitting diode; QLED, quantum dot light-emitting diode; OLED, organic light-emitting diode; PLEC, polymer light-emitting electrochemical cell; ZnS LEC, ZnS light-emitting capacitor. Adapted with permission from [3–10,12–15].

## RIGID-ISLAND APPROACH

Traditional inorganic materials such as conducting and semiconducting materials possess excellent electronic and optical properties. As a result, light-emitting diodes (LEDs) made of these materials have shown high electroluminescent performance. However, inorganic materials usually have high modulus and are brittle. Therefore, they require special structural engineering, such as rigid-island or bucked structures, in order to incorporate them into stretchable electronic devices. This is mainly because high-modulus inorganic materials easily undergo crack formation even under a small strain, resulting in internal deterioration and loss of conducting paths. In order to prevent materials from breaking, Rogers’ group designed a rigid-island-based light-emitting device with each pixel island connected by serpentine-shaped micro ribbons [3]. In their design, they fabricated the rigid LED array and connected them together with serpentine-shaped micro ribbons. Then, the entire LED array was transferred to a pre-stretched elastic substrate. After releasing the pre-strain, the serpentine-shaped micro ribbons were delaminated from the substrate to form the arch bridges. During stretching, most of the strain was released through the deformation of the serpentine-shaped micro ribbons with a maximum strain of ∼48%. Later, Samsung Electronics realized the stretchable active-matrix organic light-emitting diode (OLED) display with full colors by following a similar method [4]. Although the display resolution was reduced under deformations, a special design was employed to improve this problem. In this design, pixels were classified into many subgroups, with some pixel subgroups only being activated when the strain was loaded.

## BUCKLING APPROACH

Another strategy to achieve stretchable light-emitting devices is designing buckled structures. In this strategy, the light-emitting devices were first fabricated onto an ultra-thin flexible substrate and then this pre-fabricated device was transferred onto a pre-stretched elastomeric substrate. After releasing the elastomeric substrate, the resulting buckled structure can be obtained on the light-emitting device, which possessed the high stretchability being able to elongate up to the original flat state without any degradation. The key parameter for this device fabrication is to control the thickness of the entire light-emitting device, as a lower thickness would enable the light-emitting device to endure a lower bending radius. Based on this design, Someya's group developed the stretchable polymer light-emitting diode (PLED) with a buckled structure [5]. The entire thickness is ∼2 μm so that the resulting PLED can survive a very small radius of curvature down to several tens of micrometers combined with a maximum brightness of 120 cd/m^2^. Kim's group further used high-brightness quantum dots as the active material and pushed the device brightness up to 14 000 cd/m^2^ at 7 V [6]. However, this buckled structure may lead to the significant reduction in transmittance due to light scattering. Meanwhile, the non-planar structure is also needed to be carefully considered for further integration in practical applications.

## INTRINSICALLY STRETCHABLE POLYMER-MATRIX APPROACH

Intrinsically stretchable architectures of light-emitting devices are a promising approach for future stretchable displays. The materials required in these kinds of devices need to have low modulus and without the need for special structure designs including rigid island and buckling. These devices can be directly stretched to large strains while still maintaining good performance. To achieve low-modulus materials, light-emitting capacitor devices have been pursued. Lee's group incorporated inorganic light-emitting ZnS:Cu microparticles and silver nanowires (AgNWs) into a polydimethylsiloxane (PDMS) elastomer matrix [7]. The resulting light-emitting capacitor showed a high stretchability of 100% with a maximum brightness of 220 cd/m^2^. Shepherd's group further employed the low-modulus and stretchable hydrogel as the electrode to increase the maximum strain to >480% showed it as the electroluminescent skin of a soft robot [8]. However, due to the thicker dielectric layer and light-emitting capacitor mechanism, the driving voltage for these devices is as high as several hundred to thousand volts. The polymer light-emitting electrochemical cell (PLEC) is another type of device that is suitable for making intrinsically stretchable devices, which does not have the high-driving-voltage issue. Pei Group reported a light-emitting layer of a PLEC with a mixture of light-emitting polymer, an ionic conductor and a salt, with a maximum brightness of 300 cd/m^2^ at 12 V [9]. The brightness can be further increased to 2200 cd/m^2^ by optimizing the conductivity and transmittance of the stretchable AgNWs/polyurethane acrylate (PUA) electrode as well as the components of the light-emitting layer [10]. Due to the need for diffusion of mobile ions to form a p–i–n junction in the electroluminescent process, the response time is relatively long and a frozen junction system was studied to improve this problem [11].

On the other hand, PLEDs have high brightness, low driving voltage and short response time. Pei's group also reported an intrinsically stretchable PLED with a high brightness of 1100 cd/m^2^ by using a AgNWs/graphene oxide/PUA composite electrode [12]. However, the light-emitting conjugated polymer film in this device generated a crack at 40% strain, which significantly hindered its cycling stability during stretching. To enhance the stretchability of light-emitting polymer film, Kuo's group designed a rod–coil block copolymer exhibiting stretchability of >140% strain [13]. Another strategy to enhance stretchability was introducing a plasticizer into the light-emitting polymer film, which can reduce the film modulus by weakening the interchain interactions [14]. The maximum stretchability of the device can reach 80% strain with a maximum brightness of 2500 cd/m^2^. It is important to note that neat light-emitting conjugated polymers usually contain many electron and hole traps caused by structural defects and impurities. These traps may significantly reduce the electron and hole mobilities and also cause non-radiative trap-assisted recombination, resulting in poor electroluminescent performance. Recently, Bao's group reported a concept of introducing a specifically selected soft elastomer polyurethane into the light-emitting conjugated polymer, e.g. Super Yellow, and the resulting light-emitting layer can form nanoconfined light-emitting polymer structures through spontaneous phase separation and resulted in a charge-trapping dilution effect [15]. Due to the formation of a desirable morphology for optimal continuous charge transport and charge-trapping dilution, the electronic, optoelectronic and mechanical properties of the light-emitting layer was significantly enhanced. As a result, the resulting stretchable PLED showed a maximum device brightness of ∼7450 cd/m^2^ with a high stretchability of 100% strain.

The above results show major leaps towards the realization of next-generation stretchable displays. However, there are still many challenges for future practical applications. First, the materials and device structures need to be further improved to increase the device cycling stability under repeated stretching. Incorporation of dynamic bonds into a conjugated polymer backbone might be one of the promising strategies to improve the stretchability of light-emitting conjugated polymers [16]. Second, identifying suitable encapsulation materials to enhance the long-term stability of the device in air is becoming critically important and necessary. Finally, the resolution of current light-emitting devices is still low and fabrication processes need to be developed to achieve high-resolution and large-scale stretchable displays. Nevertheless, it is promising that high-resolution and full-color stretchable displays may become a reality in the future through the design and optimization of materials, device structures and fabrication processes. These stretchable displays would be greatly attractive to be used for human–machine interfaces (e.g. interactive 3D displays that could change shape based on the displayed contents) and biomedical applications (e.g. implantable soft displays for optogenetics and optical therapy).

## References

[1] Wang S , Xu J, Wang W et al. Nature 2018; 555: 83–8. 10.1038/nature25494 29466334

[2] Son D , Kang J, Vardoulis O et al. Nat Nanotech-nol 2018; 13: 1057–65. 10.1038/s41565-018-0244-6 30127474

[3] Kim RH , Kim DH, Xiao J et al. Nat Mater 2010; 9: 929–37. 10.1038/nmat2879 20953185

[4] Hong JH , Shin JM, Kim GM et al. Jnl Soc Info Display 2017; 25: 194–9. 10.1002/jsid.547

[5] White MS , Kaltenbrunner M, Głowacki ED et al. Nat Photon 2013; 7: 811–6. 10.1038/nphoton.2013.188

[6] Choi MK , Yang J, Kang K et al. Nat Commun 2015; 6: 7149. 10.1038/ncomms8149 25971194 PMC4479007

[7] Wang J , Yan C, Chee KJ et al. Adv Mater 2015; 27: 2876–82. 10.1002/adma.201405486 25788429

[8] Larson C , Peele B, Li S et al. Science 2016; 351: 1071–4. 10.1126/science.aac5082 26941316

[9] Yu Z , Niu X, Liu Z et al. Adv Mater 2011; 23: 3989–94. 10.1002/adma.201101986 21796688

[10] Liang J , Li L, Niu X et al. Nat Photon 2013; 7: 817–24. 10.1038/nphoton.2013.242

[11] Shao Y , Bazan GC, Heeger AJ. Adv Mater 2007; 19: 365–70. 10.1002/adma.200602087

[12] Liang J , Li L, Tong K et al. ACS Nano 2014; 8: 1590–600. 10.1021/nn405887k 24471886

[13] Jao CC , Chang JR, Ya CY et al. Polym Int 2021; 70: 426–31. 10.1002/pi.6023

[14] Kim J H , Park J W. Sci Adv 2021; 7: eabd9715. 10.1126/sciadv.abd9715 33627424 PMC7904263

[15] Zhang Z , Wang W, Jiang Y et al. Nature 2022; 603: 624–30. 10.1038/s41586-022-04400-1 35322250

[16] Oh J , Rondeau-Gagné S, Chiu YC et al. Nature 2016; 539: 411–5. 10.1038/nature20102 27853213

